# Pretreatment Neutrophil-to-Lymphocyte Ratio: A Predictor of Advanced Prostate Cancer and Biochemical Recurrence in Patients Receiving Radical Prostatectomy

**DOI:** 10.1097/MD.0000000000001473

**Published:** 2015-10-16

**Authors:** Gui-Ming Zhang, Yao Zhu, Xiao-Cheng Ma, Xiao-Jian Qin, Fang-Ning Wan, Bo Dai, Li-Jiang Sun, Ding-Wei Ye

**Affiliations:** From the Department of Urology, Fudan University Shanghai Cancer Center (GMZ, YZ, XJQ, FNW, BD, DWY); Department of Oncology, Shanghai Medical College, Fudan University, Shanghai (YZ, XJQ, FNW, BD, DWY); and Department of Urology, The Affiliated Hospital of Qingdao University, Qingdao, China (XCM, LJS).

## Abstract

The pretreatment neutrophil-to-lymphocyte ratio (NLR) is reportedly associated with the clinical outcomes of many cancers. However, it has not been widely investigated whether the pretreatment NLR is associated with the pathological characteristics of prostate cancer (PCa) and biochemical recurrence in PCa patients receiving radical prostatectomy (RP).

In this cohort study, a total of 1688 PCa patients who had undergone RP were analyzed retrospectively, and a subset of 237 of these patients were evaluated to determine the relationship between pretreatment NLR and biochemical recurrence. Patients were divided into a high-NLR group (NLR ≥2.36) and a low-NLR group (NLR < 2.36) according to the pretreatment NLR. The association between the pretreatment NLR and pathological stage and lymph node involvement was evaluated using logistic regression analysis. Time of biochemical recurrence was determined using the Kaplan–Meier method. Cox's proportional hazard regression model was used to compare the time of biochemical recurrence between the groups.

As compared with patients in the low-NLR group, those in the high-NLR group had an increased risk of pT3–4 disease (odds ratio (OR), 1.883; 95% confidence interval (CI), 1.419–2.500; *P* < 0.001), and a 1.7-fold increased risk of lymph node involvement (OR, 1.685; 95% CI, 1.101–2.579; *P* = 0.016). For the subset of 237 patients, those with a high NLR showed a significantly shorter median biochemical recurrence-free survival time (51.9 months) than those with a low NLR (76.5 months; log-rank test, *P* = 0.019). However, multivariate analysis indicated that the NLR was not an independent predictor of biochemical recurrence (hazard ratio, 1.388; 95% CI, 0.909–2.118; *P* = 0.129).

Our findings suggest that the pretreatment NLR may be associated with pathological stage and lymph node involvement in PCa patients receiving RP, and that PCa patients with a high NLR may have a higher rate of biochemical recurrence following RP than those with a low NLR.

## INTRODUCTION

As a result of altered lifestyle, lengthened life expectancy, and increased prostate-specific antigen (PSA) screening, the national burden of prostate cancer (PCa) continues to increase in China.^1^ Although patient prognosis has improved in recent years because of early detection of PCa and timely intervention, a relatively large number of patients progress to biochemical recurrence within a few months following radical prostatectomy (RP). The fact that efficient prognostic markers and models that can accurately predict PCa progression and clinical outcomes are still lacking partly contributes to the poor prognosis of PCa. Consequently, useful and reliable predictors for the facilitation of individualized risk assessment and clinical decision-making are urgently required.

Inflammation plays an important role in cancer initiation and development,^2^ and the prognostic value of inflammatory-related factors in cancer has been of paramount interest. The Glasgow prognostic score, which is based on serum C-reactive protein and albumin levels, has been shown to be a useful predictor of many cancers, including PCa.^3–6^ In addition, the neutrophil-to-lymphocyte ratio (NLR), which combines circulating neutrophil and lymphocyte counts, is one of the most common indicators of host inflammation and is often used to predict the clinical outcome of various cancers.^7–9^ In a cohort of European PCa patients, Langsenlehner et al found that increased NLR was an independent prognostic marker of progression-free survival and overall survival (OS) in PCa patients treated with 3D conformal radiotherapy.^10^ Another study group reported the association between elevated pretreatment NLR and shorter OS in patients with metastatic castration-resistant PCa (mCRPC) treated with first-line docetaxel.^11^ Furthermore, in mCRPC patients treated with second-line chemotherapy, baseline NLR was found to be associated with PSA response independent of baseline steroid use.^12^

Disease progression depends on a complex interaction between tumor characteristics and host factors. However, few studies to date have explored the correlation of pretreatment NLR with the pathological characteristics of PCa, and subsequent biochemical recurrence in patients receiving RP. The purpose of the present study was to examine the association between pretreatment NLR and the pathological features of PCa in patients who had received RP, and additionally, whether pretreatment NLR can be used as a predictor of biochemical recurrence. To our knowledge, this is the first study of its kind that has been carried out in Chinese PCa patients.

## METHODS

### Study Participants

A total of 1688 consecutive patients with pathologically confirmed PCa were enrolled in this retrospective cohort study from January 2005 to December 2014. The study was carried out at the Department of Urology in two clinical centers in eastern China: Fudan University Shanghai Cancer Center (FUSCC) and the Affiliated Hospital of Qingdao University. All patients underwent RP and standard pelvic lymphadenectomy. Patients who had received neoadjuvant therapy were excluded from the study. Data regarding age, body mass index (BMI), smoking status (ever/current or never), history of hypertension and diabetes, preoperative PSA levels, biopsy Gleason score (GS), clinical stage, prostatectomy GS and pathological stage, were compiled from medical records. Peripheral blood cell counts were performed at 1–7 days before surgery. Adjuvant hormonal therapy was administered to those patients who met the criteria according to the Guidelines for PCa established by the Chinese Urological Association. In addition, a subset of 237 consecutive patients who had received surgical treatment from January 2006 to December 2009 at the Department of Urology at FUSCC were analyzed; the objective was to determine the relationship between the pretreatment NLR and biochemical recurrence. During the follow-up, PSA was tested every 3 months. PSA >0.2 ng/mL in 2 consecutive tests is considered as biochemical recurrence. The last follow-up date was June, 2014.

The protocol was approved by the institutional research review boards of the 2 clinical centers, and written informed consent was obtained from each patient.

### Statistical Methods

NLR was calculated as the absolute neutrophil count divided by the absolute lymphocyte count (×10^9^ L^−1^). The threshold for the dichotomization of the NLR was determined to be 2.36 (high-NLR group, ≥2.36; low-NLR group, <2.36) using a receiver-operating characteristic curve and Youden's index.

Categorical variables were reported as frequencies and percentages, and the difference was compared using Pearson's *χ*^2^ tests. Unconditional logistic regression analysis was performed to calculate the odds ratio (OR) and 95% confidence interval (CI). Linear correlation analysis was used to assess the association between prognosis and NLR values. The time of biochemical recurrence was determined using the Kaplan–Meier method, and was compared using the log-rank test. Cox's proportional hazard regression model was used to evaluate the relationship between the time of biochemical recurrence and several clinical variables. All statistical tests were 2-sided, and *P* values <0.05 were considered as being statistically significant. STATA software version 12.1 (StataCorp, College Station, TX) was used for statistical analysis.

## RESULTS

This study eventually included 1229 patients with newly diagnosed PCa. Patients had a median age of 68 (range, 41–84) years and a median preoperative PSA level of 15.94 (range, 1.07–586.30) ng/mL. There were 629, 314, and 286 patients with ≤ cT2a, cT2b, and ≥cT2c disease, respectively, according to the American Joint Committee on Cancer TNM staging system (2002). Postoperative pathological evaluation determined that 780 and 449 patients had pT2 and pT3–4 disease, respectively. Furthermore, lymph node involvement was observed in 117 patients. The demographic and clinicopathological characteristics stratified by NLR group are listed in Table 1.

**TABLE 1:**
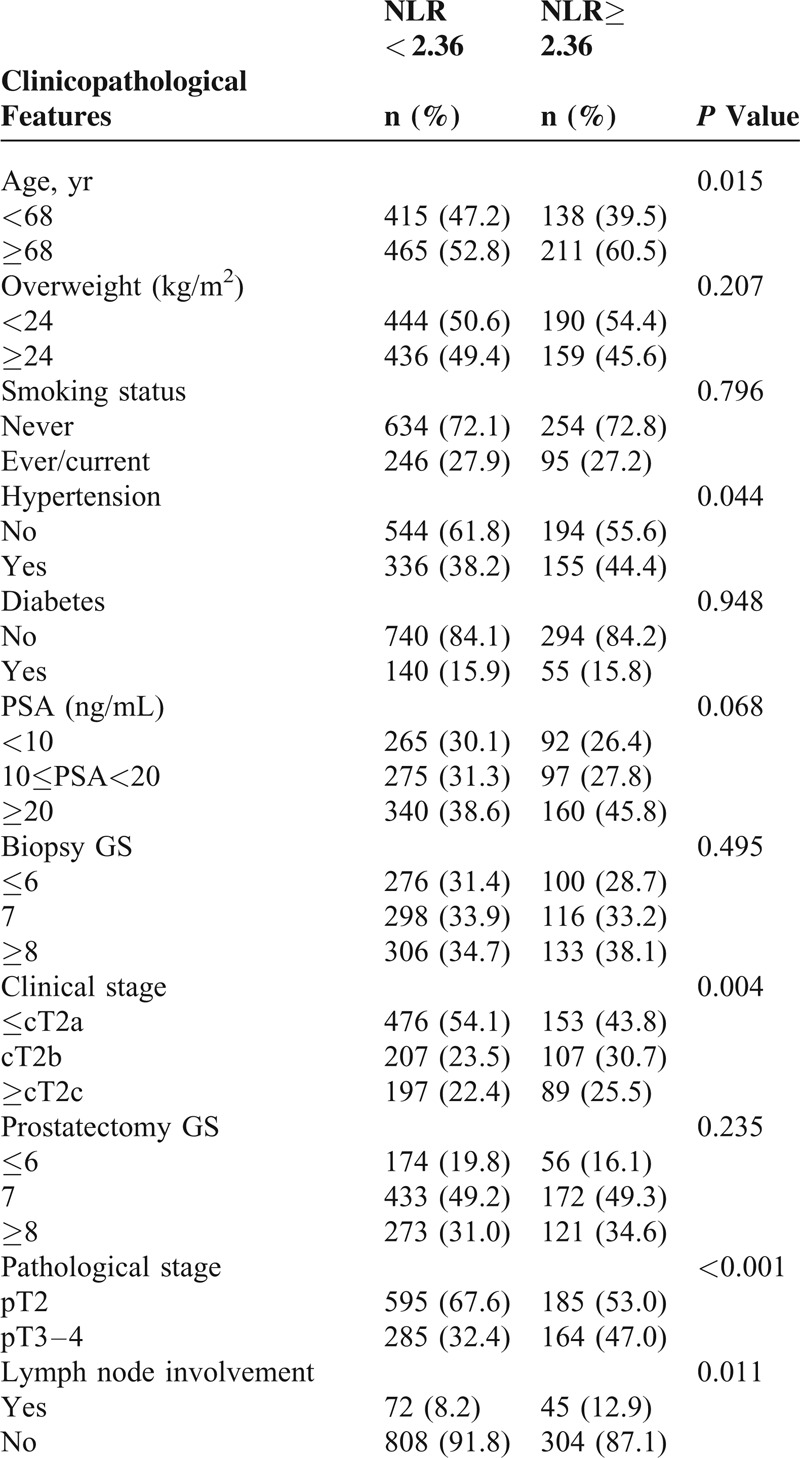
. Clinicopathological Characteristics of 1229 Prostate Cancer Patients Who Had Received Radical Prostatectomy

Peripheral blood counts indicated that the median values of white blood cell, neutrophil, and lymphocyte counts were 5.9 × 10^9^ L^−1^ (range, 3.1–15.4 × 10^9^ L^−1^), 3.3 × 10^9^ L^−1^ (range, 1.4–10.9 × 10^9^ L^−1^), and 1.8 × 10^9^ L^−1^ (range, 0.5–4.5 × 10^9^ L^−1^), respectively. There were no differences regarding obesity (BMI ≥24 kg/m^2^),^13^ smoking status, history of diabetes, PSA levels, biopsy GS, and prostatectomy GS between the groups. Patients in the high-NLR group had a higher age and less early clinical stage diseases than those in the low-NLR group. Fewer patients in the low-NLR group had a history of hypertension than those in the high-NLR group. In addition, relative to the patients in low-NLR group, patients in the high-NLR group had a higher incidence of pT3–4 disease and greater lymph node involvement (Table 1).

Next, the association between postoperative pathological stage and lymph node involvement, and preoperative PSA levels, biopsy GS, clinical stage, and NLR was assessed using univariate and multivariate logistic regression models. After adjustment for potential confounders, an NLR ≥2.36 was associated with a 1.8-fold increased risk of pT3–4 disease (OR, 1.883; 95% CI, 1.419–2.500), and was found to be an independent predictor of lymph node involvement (OR, 1.685; 95% CI, 1.101–2.579) (Table 2).

**TABLE 2:**
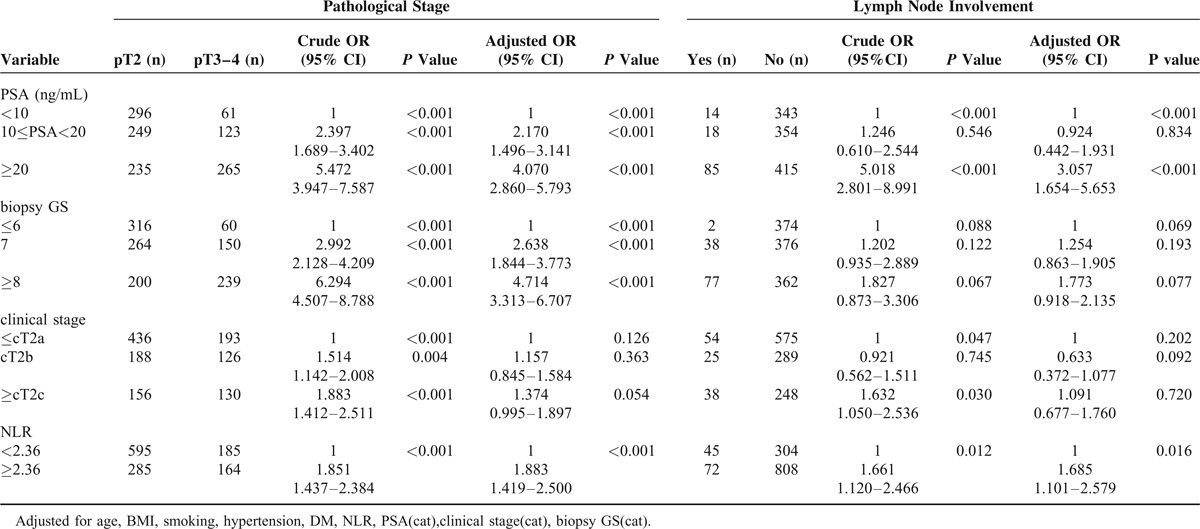
Logistic Regression Analysis of the Association between Pretreatment NLR and Pathological Features in Prostate Cancer Patients

The demographic and clinicopathological characteristics of 237 patients in whom the relationship between NLR and biochemical recurrence was evaluated are detailed in Table 3. Similar to the whole group of patients, patients with an NLR ≥2.36 presented with more pT3–4 disease and more lymph node involvement. Comparison of other variables indicated no differences between the high-NLR and low-NLR groups.

**TABLE 3:**
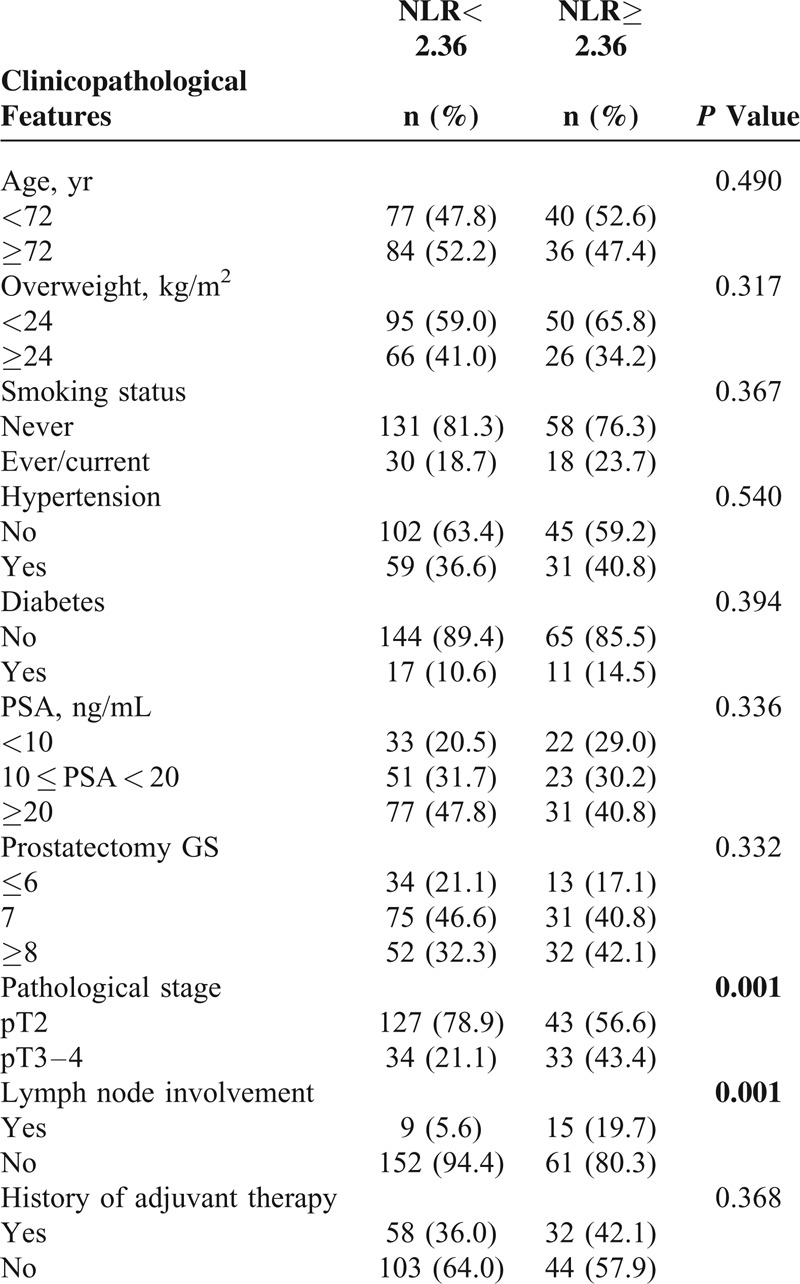
Clinicopathological Characteristics of a Subset of 237 Prostate Cancer Patients Who Had Received Radical Prostatectomy

**FIGURE 1:**
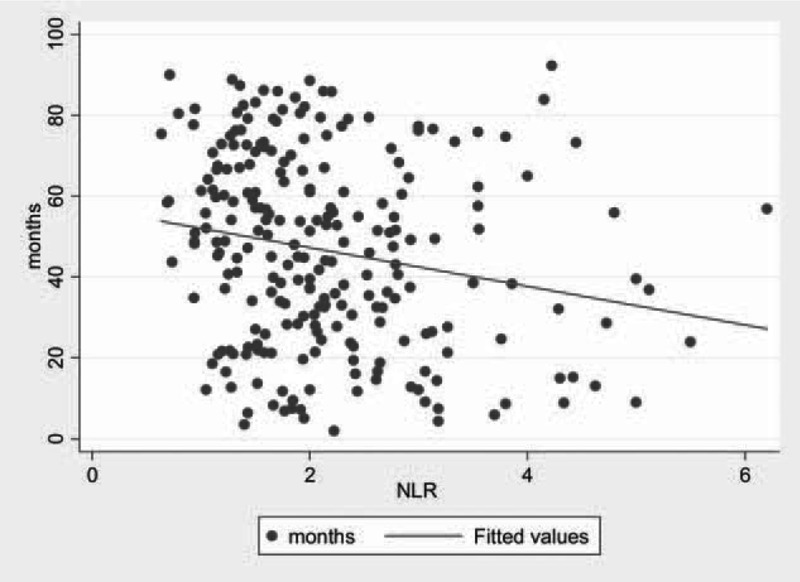
Linear correlation analysis of the association between prognosis and NLR values.

**FIGURE 2:**
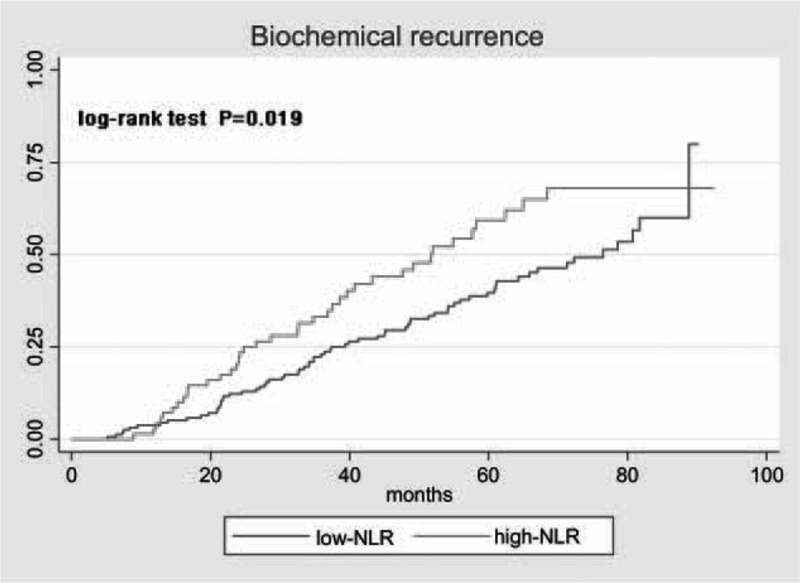
Kaplan–Meier curves for time of biochemical recurrence in prostate cancer patients receiving radical prostatectomy.

For the subset of 237 patients, the mean follow-up time was 46.6 months. Loss of follow-up and death occurred in 21 and 3 patients, respectively. Patients in the high-NLR group showed higher white blood cell (median values: 6.6 × 10^9^ L^−1^ vs. 5.7 × 10^9^ L^−1^, *P* *<* 0.001) and neutrophil counts (median values: 4.4 × 10^9^ L^−1^ vs. 3.1 × 10^9^ L^−1^, *P* *<* 0.001), as well as lower lymphocyte counts (median values: 1.4 × 10^9^ L^−1^ vs. 1.9 × 10^9^ L^−1^, *P* *<* 0.001). The median biochemical recurrence-free survival time was 65.0 months. The prognosis of patients was significantly associated with NLR values (*P* = 0.002) (Figure 1). Kaplan–Meier curves for biochemical recurrence probability are presented in Figure 2. In the high-NLR group, the median biochemical recurrence-free survival time (51.9 months) was significantly shorter (log-rank test, *P* = 0.019) than in the low-NLR group (76.5 months). However, further Cox's proportional hazard regression analyses indicated that after adjusting for confounding variables, such as age, BMI, smoking status, history of hypertension and diabetes, PSA levels, prostatectomy GS, pathological stage, lymph node involvement and history of adjuvant therapy, NLR was not an independent predictor of biochemical recurrence (hazard ratio [HR], 1.388; 95% CI, 0.909–2.118; *P* = 0.129; Table 4).

**TABLE 4:**
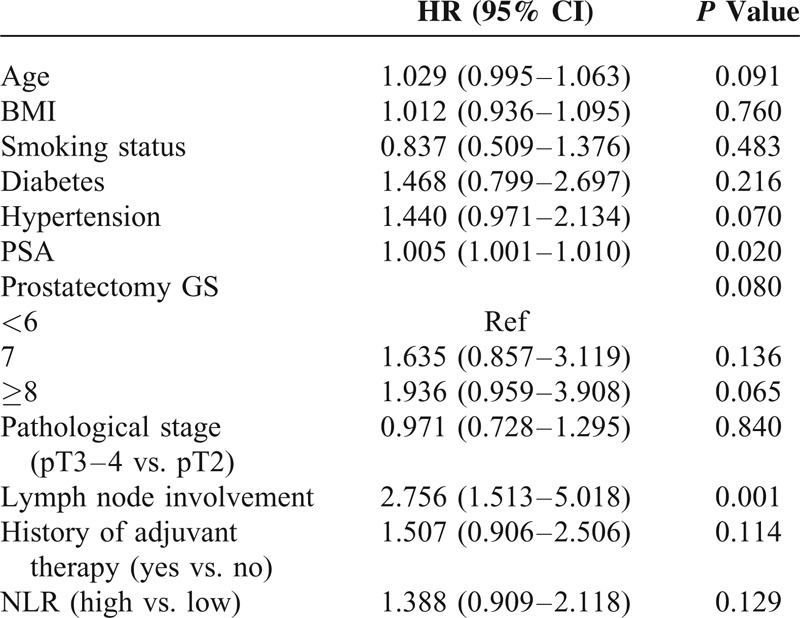
Multivariate Analysis of Predictors of Time of Biochemical Recurrence in Prostate Cancer Patients Who Received Radical Prostatectomy

## DISCUSSION

In this retrospective cohort study conducted in eastern China, we found that high pretreatment NLR was significantly associated with pT3–4 disease and lymph node involvement in PCa patients who had received RP. In addition, we revealed a favorable prognosis in patients with a low pretreatment NLR, although it was not found to be an independent predictor of biochemical recurrence.

The interaction between inflammation and tumors has been studied in detail over the past decades. Through multiple mechanisms, inflammation is involved in carcinogenesis in all disease stages.^14^ For example, inflammatory cells can stimulate proliferative signaling, induce resistance to apoptosis, maintain DNA mutations, and promote angiogenesis, invasion, and metastasis through the secretion of a series of bioactive molecules.^2^ Host immunity, to a certain extent, is thought to reflect the microenvironment either in favor of or against tumor development, and subsequent oncological outcomes. To date, several measurements of systemic inflammatory response, such as NLR, platelet-to-lymphocyte ratio, C-reactive protein, and serum albumin, have been investigated in cancer patients. Despite inconsistent results, these markers reportedly have valuable diagnostic and prognostic roles. As an important inflammatory marker, the NLR has been extensively studied regarding the assessment of risk stratification and prognosis in various cancers, including PCa. Templeton et al. discovered and further validated the prognostic role of the pretreatment NLR in mCRPC patients.^15^ They found that an NLR >3 was associated with worse OS and was an independent indicator of poor prognosis.^15^ Similar results have been reported by Nuhn et al.^11^ However, another group found that the NLR could only be used to predict PSA response, but not clinical outcomes in mCRPC patients treated with docetaxel.^16^ Gazel and his colleagues reported that PCa patients with a high NLR had higher rates of PSA recurrence following RP than those with a low NLR.^17^ Similarly, in our study, a positive correlation was observed between high NLR and elevated biochemical recurrence rates. These inconsistent conclusions might be partly ascribed to the discrepancy in study design, patient ethnic background and characteristics, as well as different NLR cutoffs. For example, the baseline PSA levels in our study were remarkably higher than those in Western countries. Additionally, because there has been no unified standard cutoff to date that can be applied in otherwise similar studies, several NLR cutoffs have been used to stratify study groups in the different studies. However, for the first time, we have found that a high pretreatment NLR was associated with advanced PCa pathological stage and lymph node involvement. Advanced pathological stages and lymph node metastases are confirmed risk factors for disease progression in PCa. Consequently, we speculate that the effect of pretreatment NLR on biochemical recurrence might in part be the result of the association with advanced pathological features. Clearly, further validations including large-scale studies involving patients from different races and agreed-upon standards are needed; in addition, the exact way in which NLR correlates with PCa progression remains to be elucidated.

Being important components of the host immune system, peripheral leukocytes can greatly influence tumor development and progression. As a result of increasing tumor burden, activated neutrophils can infiltrate tumor tissues and promote tumor metabolism by secreting a variety of bioactive molecules, such as vascular endothelial growth factor and reactive oxygen species.^18,19^ Conversely, lymphocytes can exert an anti-tumor effect by inhibiting tumor cell proliferation and migration, inducing tumor cell apoptosis and mediating antibody-dependent cell-mediated cytotoxicity.^20,21^ It has been reported that low absolute lymphocyte counts are correlated with an immunosuppressive status in many cancers, which is thought to be associated with impaired patient survival.^22^ Therefore, NLR, a combination of neutrophils and lymphocytes, may represent a balance in host immunity against malignancy, and can be regarded as a simple and useful monitor during tumor follow-up.

The present study had several limitations. First, data regarding all patients were reviewed retrospectively, which entailed an intrinsic selection bias. Second, some important information, such as family history, medication usage, and C-reactive protein, was not gathered. These potential confounders might limit the statistical power and affect the results of this study. Third, peripheral blood cell counts were performed only one time, which might also cause bias. Fourth, owing to concomitant infection or drug usage, NLR might differ among individuals, yet these patients were not excluded from the study and was not accounted for in the results. Finally, the current study involved Chinese men and thus cannot be generalized to other ethnic populations. In addition, the cross of survival curve at the end of follow-up might be caused by the following reasons: first, with longer follow-up, fewer patients were at risk of biochemical recurrence, and exceptional survival in one patient may influence the curve of high-NLR group; second, NLR may indicate a short time to recurrence other than the probability of recurrence. Therefore, large patient samples with longer follow-up were needed to better define the prognostic role of NLR in PCa.

In conclusion, our findings suggest that an increased pretreatment NLR may be associated with pT3–4 disease and lymph node involvement in PCa patients who had received RP. Furthermore, PCa patients with a high NLR may have a higher rate of biochemical recurrence following RP than those with a low NLR. As NLR is a cost-effective and easily measurable monitor, and shows favorable prognostic value, this marker warrants further validation in more patients and clinical practice.
